# Self-stigma among people with crack cocaine and alcohol dependence: a comparative study

**DOI:** 10.1186/1940-0640-8-S1-A63

**Published:** 2013-09-04

**Authors:** Pollyanna Santos da Silveira, Ana Luisa M Casela, Érika P Monteiro, Gabriela CL Ferreira, Jéssica VT Freitas, Leonardo F Martins, Telmo Mota Ronzani, Ana Regina Noto

**Affiliations:** 1 Center for Research, Intervention, and Evaluation for Alcohol and Drugs (CREPEIA), Federal University of Juiz de Fora, Brazil, Juiz de Fora, Brazil; 2 Department of Social Psychology and Public Health, Federal University of Juiz de Fora, Juiz de Fora, Brazil

## 

Self-stigma represents a barrier in finding appropriate treatment services, and brings several negative consequences for the individual and the worsening of the situation. Considering that substance dependence is one of the most stigmatizing health conditions, this study aims to assess self-stigma among crack, alcohol, and both substances (crack and alcohol) dependents. The instruments used were: 1) sociodemographic questionnaire to describe participants’ characteristics; the Internalized Stigma of Mental Illness (ISMI) scale adapted for substance users to assess self-stigma; 3) MINI to confirm the substance dependence diagnosis. The sample was composed by 461 patients divided into three groups: 165 alcohol dependents, 141 crack dependents, and 155 crack and alcohol dependents. Most participants were male (91.4%), unemployed (68.6%), and single (70.5%), with incomplete elementary school education (53.3%), with a mean age of 35.38 years. Regarding to involvement in illicit activities, 56.8% stated they were not involved with this type of activity. Comparing self-stigma among the groups investigated, it was found a significant difference between the groups regarding the ISMI overall scores. Similarly, ISMI subscales Alienation, Discrimination Experience, Social Withdrawal and Stigma Resistance also showed a significant difference between groups. On the other hand, the degree of agreement with stereotypes measured by the Stereotype Endorsement subscale did not differ between the groups investigated. The negatives stereotypes associated with substance dependence are equally endorsed and internalized by all groups, however this study showed evidence that crack dependents internalize more negative evaluations related to their condition, and therefore are less likely to seek treatment and adhere to it. The results pointed out the need to consider the impact of stigma on prevention and treatment strategies, in order to encourage patients to seek help and ensure the benefits of interventions. Funding: Fapesp, Capes, CNPQ.

